# The therapeutic potential of Myoki, a novel peptide in muscle atrophy: mechanisms and applications

**DOI:** 10.3389/fphar.2026.1663850

**Published:** 2026-02-12

**Authors:** Eun Mi Kim, Seon Soo Kim, Yong Geon Hyun, Su Yeon Lee, Yong Ji Chung

**Affiliations:** Research and Development Department, Caregen Co., Ltd., Seoul, Republic of Korea

**Keywords:** muscle atrophy, muscle hypertrophy, myogenic regulatory factors, myostatin, protein degradation, protein synthesis, sarcopenia

## Abstract

**Background:**

With the global population rapidly aging, the prevalence of sarcopenia, referring to a progressive loss of skeletal muscle mass and strength, is increasing, highlighting the need for effective preventive and therapeutic strategies. In this study, we evaluated the efficacy of Myoki, a synthetic peptide, in muscle atrophy models *in vitro*, *in vivo*, and in a human clinical trial.

**Methods:**

This study evaluated the therapeutic potential of Myoki, a synthetic peptide, in preventing muscle atrophy by assessing its effects in multiple models. *In vitro*, C2C12 myoblasts were used to evaluate Myoki’s impact on cytotoxicity, myotube differentiation, and muscle atrophy in DEX-induced atrophy models, with effects measured by western blot, qRT-PCR, and immunofluorescence. The binding affinity between Myoki and myostatin was further evaluated using surface plasmon resonance (SPR) and confirmed by ELISA-based assays. *In vivo*, the accelerated aging mouse model (SAMP8) was employed to investigate Myoki’s effect on muscle fiber area, collagen deposition, and muscle atrophy markers, with muscle histology, fibrosis, and fluorescence intensities of key proteins assessed via immunofluorescence after 45 weeks of treatment. In the clinical study, a randomized, double-blind, placebo-controlled trial was conducted with 80 patients diagnosed with muscle atrophy, who received Myoki (200 mg/day) or placebo for 12 weeks. Key efficacy endpoints included changes in muscle mass (DEXA), handgrip strength, walking speed (6-m walk test), and serum marker levels, with safety monitored throughout the trial.

**Results:**

Myoki demonstrated no cytotoxicity up to 500 μM in C2C12 cells and significantly promoted myotube differentiation. In DEX-induced muscle atrophy models, Myoki restored protein synthesis signaling and reduced muscle degradation pathways. In the SAMP8 mouse model, Myoki improved muscle fiber area, reduced collagen deposition, and mitigated muscle fibrosis. In the clinical trial, Myoki supplementation for 12 weeks significantly improved muscle mass, walking speed, and grip strength, along with favorable changes in serum markers related to muscle growth and damage.

**Conclusion:**

Our results position Myoki as a promising potential candidate for mitigating muscle atrophy, including age-associated muscle loss. These findings support its therapeutic potential for conditions such as sarcopenia, making it a valuable candidate for further clinical exploration.

**Clinical Trial Registration:**

clinicaltrials.gov, CTRI/2024/01/061919.

## Graphical Abstract

**Figure:**
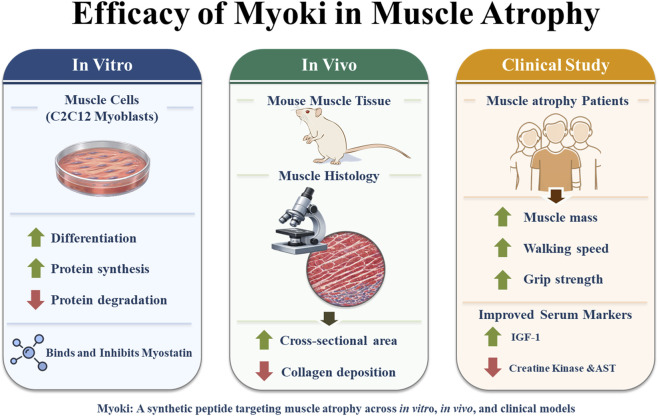

## 1 Introduction

With the aging global population, sarcopenia has emerged as a significant public health concern (Beaudart et al., 2014). Characterized by the gradual loss of muscle mass and function, sarcopenia primarily affects older adults (Zhang et al., 2024; Cruz-Jentoft et al., 2019), and increases the risk of falls, fractures, disability, and mortality, significantly reducing the quality of life of the patients (Beaudart et al., 2017). Therefore, maintaining muscle mass and function is critical to healthy aging.

The pathophysiology of sarcopenia has not yet been fully elucidated, and growing body of evidence suggests its association with multifactorial mechanisms, including impaired protein homeostasis, across diverse physiological and pathological conditions. Sarcopenia-related muscle atrophy is accompanied by reduced myofiber diameter, which could ultimately lead to reduced muscle mass and functional decline. Although an imbalance between protein synthesis and degradation is an important contributor to muscle atrophy, muscle loss progresses through the combined effect of multiple mechanisms (Wang et al., 2025; Sandri, 2008; Bodine et al., 2001a). Beyond protein turnover, impaired satellite cell proliferation and self-renewal might limit regenerative capacity (Yablonka-Reuveni, 2011; Li et al., 2025), and mitochondrial/metabolic dysfunction could further compromise myofiber maintenance and myogenesis (Sartorelli and Ciuffoli, 2024). In addition, systemic and lifestyle-related factors (e.g., anabolic hormone level alterations, inflammation- or disease-driven catabolic stimuli, insufficient physical activity, and inadequate protein intake) might contribute to muscle loss (Sgrò et al., 2019; Ji et al., 2022). Therefore, to delay the progression of sarcopenia, understanding the mechanisms underlying muscle loss and developing targeted therapeutic strategies are essential.

The muscle regeneration process starts with satellite cell activation, followed by myoblast proliferation and differentiation, leading to mature myotube formation. Myoblast differentiation and mature myotube formation are regulated by myogenic regulatory factors (MRFs), including MyoD, Myf5, MyoG (Myogenin), and Myf6 (Zammit, 2017; Segales et al., 2015), representing key transcription factors upregulated during myogenesis and critical for directing stem cell differentiation into myotube cells. Ultimately, differentiated myotube cells might fuse to form new muscle fibers or integrate with existing ones. Newborn mice reportedly lack MyoD and Myf5, exhibiting a severe myoblast and muscle fiber deficiency (Rudnicki et al., 1993).

Myostatin is a key negative regulator of skeletal muscle growth. Alterations in myostatin expression have been described in human sarcopenia, suggesting that myostatin might represent a therapeutic target linked to muscle loss (Rodriguez et al., 2014; Léger et al., 2008). Genetic or pharmacological inhibition of myostatin reportedly induces muscle hypertrophy, whereas myostatin overexpression reportedly promotes muscle atrophy (Trendelenburg et al., 2009; Elkina et al., 2011). These effects are mediated, in part, by reduced protein synthesis and the enhanced activation of protein degradation pathways, including the suppression of the Akt/p70S6K signaling axis and the activation of the ubiquitin–proteasome system (Trendelenburg et al., 2009; Elkina et al., 2011). Consistently, myostatin upregulates ubiquitin E3 ligases (e.g., atrogin-1/MAFbx and MuRF-1), thereby accelerating muscle protein degradation (Lokireddy et al., 2011; McFarlane et al., 2006). In line with the therapeutic potential of this pathway, myostatin-deficient cattle and knockout mice exhibit a marked “double-muscle” phenotype (McPherron and Lee, 1997; Grobet et al., 1997), and multiple anti-myostatin strategies reportedly improve muscle mass and strength (Bogdanovich et al., 2002). Taken together, these results support that myostatin inhibition is a promising approach to induce muscle-wasting conditions.

Accordingly, we sought to identify peptide candidates that could counteract myostatin-associated catabolic signaling and muscle atrophy, we thus conducted preliminary screening of multiple peptide groups and identified Myoki, a synthetic peptide, as a lead candidate. We selected Myoki as it promoted myotube differentiation in C2C12 cells and attenuated the dexamethasone (DEX)-induced activation of muscle protein degradation signaling, particularly E3 ubiquitin ligase atrogin-1/MAFbx upregulation (Supplementary Figure S1). In this study, we evaluated how Myoki affects skeletal muscle atrophy and investigated its underlying mechanisms using C2C12 myoblasts and an accelerated aging mouse model. We further assessed the clinical efficacy and safety of using Myoki in patients with clinically diagnosed muscle atrophy (ICD-10-CM Code: M62.5) to validate its therapeutic potential.

## 2 Materials and methods

### 2.1 Synthesis and purification of Myoki peptide

Myoki was synthesized by Fmoc-based solid-phase peptide synthesis using a chlorotrityl chloride (2CTC) resin, swollen in dichloromethane (DCM) under agitation, then filtered. The first amino acid was dissolved in DCM, combined with N,N-diisopropylethylamine (DIPEA), and added to the reaction vessel for 2 h to allow for loading onto the resin. After filtration and washing, coupling completion was verified, and the Fmoc protecting group was removed using 20% piperidine in N,N-dimethylformamide (DMF). Subsequent amino acids were coupled using 2-(1H-benzotriazol-1-yl)-1,1,3,3-tetramethyluronium hexafluorophosphate (HBTU) in DMF with DIPEA. The coupling and Fmoc deprotection steps were repeated sequentially until the final amino acid was incorporated, yielding the desired peptidyl resin. The completed peptidyl resin was cleaved and globally deprotected using a cleavage cocktail (TFA:TIS:PW:EDT = 94:1:2.5:2.5). The crude peptide was precipitated/recrystallized with methyl tert-butyl ether (MTBE) to recover peptide powder, which was further purified by column chromatography using C18 resin (YMC CO., LTD., Japan) to obtain high-purity Myoki.

### 2.2 HPLC and LC–MS/MS analysis

Peptide purity was assessed using high-performance liquid chromatography (HPLC; U-3000, Thermo Fisher Scientific, USA) with a C18 column (250 × 4.65 mm, 5 μm; Pursuit XRs, Agilent Technologies, USA). Molecular weight was determined using liquid chromatography–mass spectrometry (LC–MS/MS) (3200 Q-trap, AB SCIEX, USA) with the following MS/MS conditions: ESI positive mode; curtain gas: 20; collision gas: high; ionspray voltage: 5,500; temperature: 350 °C; ion source gases 1 and 2 at 50. The compound settings comprised declustering and entrance potentials, collision energy, and collision energy spread of 50–80, 10, 10–50, 1–10, respectively. The Myoki peptide (sequence EAEYEHL, 7-mer) yielded a purity and molecular weight of 99.8% and 889.9 Da, respectively.

### 2.3 Cell culture

The C2C12 myoblasts used in this study were purchased from ATCC (CRL-1772™, Manassas, VA, USA). The cells were cultured in growth medium (GM), consisting of Dulbecco’s Modified Eagle Medium (DMEM, 25 mM glucose) supplemented with 10% fetal bovine serum (FBS) and 1% penicillin streptomycin (all obtained from Thermo Fisher Scientific). All cells were incubated in a humidified atmosphere at 37 °C with 95% air and 5% CO_2_.

### 2.4 Cytotoxicity assay

To assess Myoki cytotoxicity in undifferentiated C2C12 myoblasts, an assay using EZ-Cytox (DoGenBio, Seoul, Korea) was performed. First, C2C12 myoblasts were seeded at a density of 1 × 10^4^ cells per well in a 96-well culture plate and incubated for 24 h at 37 °C in 5% CO_2_ in DMEM supplemented with 10% FBS. Next, the cells were treated at various Myoki concentrations (3.9, 7.81, 15.63, 31.25, 62.5, 125, 250, and 500 μM) and cultured for an additional 24 h. Subsequently, 10 μL of EZ-Cytox reagents were added to each well, followed by a 1-h incubation. The absorbance was measured at 450 nm using a microplate reader, and the relative cell viability was estimated based on the absorbance of the untreated control group, considered as 100% viability.

### 2.5 Cell differentiation and muscle atrophy model

To induce C2C12 myotube differentiation, myoblasts were seeded in 6-well plates at 5 × 10^5^ cells per well. Once reaching 90% confluency, they were cultured in DMEM supplemented with 2% horse serum (Thermo Fisher Scientific) and 1% penicillin-streptomycin. To evaluate the effect of Myoki, the cells were co-cultured with 5, 50, or 100 μM Myoki for 3 or 6 days. Differentiation was observed using an optical microscope (LEICA, 100×). Myotube morphology (length and diameter) was quantified in ImageJ (NIH) using the same images: myotube length was measured by tracing the long axis of each myotube (end-to-end), and myotube diameter was measured as the short-axis width at three positions along each myotube; the mean of the three width values was recorded as the diameter. For each experimental group, three randomly captured images (fields) were analyzed, and at least 15–20 myotubes per image were quantified under identical imaging settings. For the muscle atrophy model, cells were similarly seeded and differentiated for 5 days, then treated for 24 h with 10 μM dexamethasone (DEX, Sigma-Aldrich) combined with 10 or 100 μM Myoki.

### 2.6 Indirect immunofluorescence in C2C12 cells

Differentiated C2C12 cells cultured on coverslips were fixed at room temperature for 10 min with PBS containing 3.7% paraformaldehyde. Next, the samples were permeabilized with 0.25% Triton X-100/PBS for 10 min, blocked with 1% BSA/PBS, and incubated overnight with primary antibodies, anti-MyoD (Santa Cruz, sc-377460) and anti-MYH3 (Santa Cruz, sc-53091), followed by washing with PBS. The samples were then incubated for 1 h Alexa Fluor 488–conjugated goat anti-mouse IgG H&L (abcam, ab150113). Nuclei were stained with 4′,6-diamidino-2-phenylindole (DAPI; blue) for 5 min. The coverslips were mounted onto glass slides using an aqueous mounting solution (Biomeda, Foster City, CA, USA) and left to dry. Images were captured using a confocal laser-scanning microscope (LEICA, Wetzlar, Germany) at ×200 and ×400 magnifications for MYH3 and MyoD, respectively. The fluorescence intensity of each marker was quantified using ImageJ. For fusion index analysis, MYH3-stained images were used to quantify myotube formation by counting the number of DAPI-positive nuclei located within MYH3-positive multinucleated myotubes (defined as MYH3-positive cells containing ≥2 nuclei) and the total number of nuclei per field. Fusion index (%) was calculated as (nuclei within MYH3-positive multinucleated myotubes/total nuclei) × 100. Fusion index was calculated from multiple randomly selected fields captured under identical imaging settings and quantified in ImageJ.

### 2.7 RT-PCR and RT-qPCR assay

To investigate the regulatory effects of Myoki on the expression of differentiation-related genes (Myf5, Myf6, MyoD, and MyoG) in C2C12 cells, reverse transcription PCR (RT-PCR) and real time quantitative PCR (RT-qPCR) were performed. Total RNA was extracted, and complementary DNA (cDNA) samples were synthesized using a cDNA reverse transcription kit (Enzynomics, Daejeon, South Korea) according to the manufacturer’s instructions, using a PCR thermal cycler (Eppendorf, Hamburg, Germany). For RT-PCR, the PCR products were electrophoresed in 1.5% agarose gel containing EtBr at 100 V for 30 min and visualized using a ChemiDoc™ XRS imaging system (Bio-Rad, Hercules, CA, USA). RT-qPCR was performed using SYBR Green PCR Master Mix (Applied Biosystems, Foster City, CA, USA) for quantitative analysis. Glyceraldehyde-3-phosphate dehydrogenase (GAPDH) was used as a housekeeping gene for normalization. Relative quantification (RQ) was calculated using the ΔΔCt method, and data were expressed as RQ values by comparing the threshold cycle (Ct) of each target gene to that of GAPDH. The linearity and amplification efficiency of the PCR reactions were evaluated using serial dilutions of cDNA, and all reactions were performed in triplicate. The primer sequences used were as follows: Myf5 forward (5′-TAT GAA GGC TCC TGT ATC CC-3′), Myf5 reverse (5′-ACG TGC TCC TCA TCG TCT G-3′), Myf6 forward (5′-TGC TAA GGA AGG AGG AGC AA-3′), Myf6 reverse (5′-CCT GCT GGG TGA AGA ATG TT-3′), MyoD forward (5′-AGT GAA TGA GGC CTT CGA GA-3′), MyoD reverse (5′-CTG GGT TCC CTG TTC TGT GTA-3′), MyoG forward (5′-ACC AGG AGC CCC ACT TCT AT-3′), MyoG reverse (5′-ACG ATG GAC GTA AGG GAG TG-3′).

### 2.8 Western blot assay

Treated C2C12 cells (5 × 10^5^ cells/well) were harvested in lysis buffer (Sigma-Aldrich, 2910) and centrifuged at 12,000 g for 10 min; proteins in the supernatant were collected. Protein concentrations were measured using the BCA Protein Assay Kit (Thermo Fisher Scientific). Each 20 μg sample was separated using 10% SDS–PAGE and transferred onto a polyvinylidene fluoride membrane (Millipore, Burlington, MA, USA). Non-specific binding was blocked with 5% non-fat dry milk for 1 h. The membranes were incubated overnight with primary antibodies: Anti-Myf5 (Abcam, ab125078), Anti-Myf6 (Abcam, ab182842), Anti-MyoD (Santa Cruz, sc-377460), Anti-MyoG (Santa Cruz, sc-52903), Anti-α-actinin (Santa Cruz, sc-17829), Anti-MYH3 (Santa Cruz, sc-53091), Anti-p-mTOR (Ser2448) (CST, #5536), Anti-p-Akt (Ser473) (CST, #9271), Anti-p-p70 S6 Kinase (Thr389) (CST, #9205), Anti-myostatin (Abcam, ab124721), Anti-atrogin-1/MAFbx (Santa Cruz, sc-166806), and Anti-MuRF1 (Santa Cruz, sc-398608). Membranes were then incubated for 1 h with HRP-conjugated secondary antibodies. Blot images were captured with the ECL system (Amersham, UK) and quantified using ImageJ.

### 2.9 Surface plasmon resonance (SPR) analysis

The Myoki–Myostatin interaction was measured as SPR using the Biacore T200 system (Cytiva). The human recombinant Myostatin-Fc protein (Sino Biological, #50441-M01H) was immobilized on the surface of a CM5 sensor chip at 2210.3 response units (RUs) using an EDC/NHS amine coupling kit, following the manufacturer’s instructions. The Myoki peptide was injected into the flow cell at a rate of 30 μL/min at 25 °C, using HBS-EP buffer as the running buffer. Data analysis was performed using Biacore T200 Evaluation Software version 3.1 (Cytiva).

### 2.10 Enzyme-linked immunosorbent assay (ELISA)-Based receptor binding assay

Myoki, diluted in coating buffer (R&D Systems, #DY006), and activin R2B (ASRO Biosystem, #ACB-H82E3) were dispensed into ELISA plates (Thermo Fisher Scientific, #439454) and coated overnight at 4 °C. Plates were washed with PBST and blocked with 3% (w/v) BSA for 2 h at room temperature. They were then incubated for 2 h with Fc gamma-tagged myostatin/GDF-8 (Sino Biological, #50441-M01H) diluted in PBST. After another PBST wash, plates were treated with HRP-linked Anti-IgG Fc gamma (CST, #32935S, 1:1,000) for 2 h. Finally, color was developed with TMB (Sigma, #T0440), the reaction stopped with 1 M H_2_SO_4_, and absorbance was measured at 450 nm.

### 2.11 Animal study design

Eight week old male senescence-accelerated mouse-prone 8 (SAMP8)/Ta Slc and senescence-accelerated mouse-resistant 1 (SAMR1)/Ta Slc mice (weighing 30 ± 2 g) were purchased from Central Lab Animal Inc. (Seoul, Korea) and housed at 24 °C in cages under a 12/12 h light/dark cycle with *ad libitum* access to food and water. After a 7days acclimation period, they were randomly assigned into three groups (n = 5 per group): a normal aging control group (SAMR1), an accelerated aging group (SAMP8), and a Myoki treatment group (Myoki + SAMP8). Mice in all groups were dosed once daily by oral gavage for 45 weeks. Control and negative control group mice received 200 µL PBS, whereas Myoki group mice received 20 mg of Myoki dissolved in 200 µL PBS. This study was approved by the Institutional Animal Care and Use Committee (approval number CG-R22-001).

### 2.12 Histopathological image analysis

Quadriceps (Quad) and gastrocnemius (Gas) muscle tissues were harvested and immersion-fixed in 10% neutral buffered formalin at 4 °C for 24 h. Following fixation, the tissues were dehydrated through a graded series of ethanol, cleared in xylene, and embedded in paraffin. Paraffin-embedded tissues were sectioned at a thickness of 5 μm. The tissue sections mounted on slides were deparaffinized, washed, and stained with hematoxylin (Dako, S3309) and eosin (Thermo Fisher Scientific, #6766007) (H&E). To assess the degree of muscle fibrosis, they were also stained using a Sirius Red Stain Kit (Abcam, ab150681) according to the manufacturer’s instructions. Both H&E and Sirius Red-stained slides were observed at ×200 magnification using an optical microscope (LEICA). For each animal, three images were randomly selected from the stained sections, and the total stained area per image, rather than individual muscle fibers, was quantified using ImageJ software.

### 2.13 Indirect immunofluorescence in skeletal muscle tissues

Muscle tissues fixed in formalin were sectioned into 5-μm paraffin section. They were treated with heat-mediated antigen retrieval in TRS buffer TRS buffer (pH 6.2) and peroxide-blocked using reagent (Epredia, TA-125-PBQ). Primary antibodies—anti-myostatin (Abcam, ab124721), anti-atrogin-1/MAFbx (Santa Cruz, sc-166806), and anti-fast MyHC (Abcam, ab91506)—were diluted 1:250 or 1:500 in anti-body diluent (GBI Labs, E09-300) and incubated overnight at 4 °C. After PBS washing, Samples were incubated for 1 h with goat Alexa Fluor 488–conjugated goat anti-mouse IgG H&L and anti-rabbit IgG H&L (abcam, ab150113, ab150077). Nuclei were stained with 4′,6-diamidino-2-phenylindole (DAPI; blue) for 20 min, mounted using Aqua Mounting Media (Biomeda), and imaged at 200× with a confocal fluorescence microscope (LEICA, Wetzlar, Germany). Fluorescence intensities were quantified using ImageJ software, and results were statistically analyzed.

### 2.14 Study design and participants

This study was a randomized, double-blind, placebo-controlled, parallel-group clinical trial designed to evaluate the efficacy and safety of Myoki peptide in patients with muscle atrophy (ICD-10-CM code: M62.5; muscle wasting and atrophy, not elsewhere classified). The study was conducted at Biosite Research Private Limited (Shanthipura, India) under the New Drugs and Clinical Trials Rules (2019) issued by CDSCO (Ministry of Health and Family Welfare, Government of India), and in compliance with GCP, applicable local regulatory requirements, the Declaration of Helsinki (Brazil 2024), ICMR guidelines (2017), and ICH-GCP E6 (R2). Ethical approval was obtained from the Excel Hospital Institutional Ethics Committee (ECR/1670/Inst/TG/2022) and ethics committees of other participating centers, and the trial was prospectively registered (CTRI/2024/01/061919). All participants provided written informed consent prior to any study procedures. Key eligibility criteria included adults aged ≥18 years with low muscle mass by DEXA (men: <7.0 kg/m^2^; women: <5.4 kg/m^2^) and either low handgrip strength (men: <28 kg; women: <18 kg) or low physical performance (6-m walk speed <1.0 m/s). Major exclusion criteria included uncontrolled systemic disease, musculoskeletal or neurological disorders, recent anabolic steroid use, and concomitant medications known to affect protein metabolism; full inclusion and exclusion criteria are provided in Supplementary Table S1. Of 89 patients screened, seven were screen failures and two withdrew prior to randomization; 80 eligible participants were enrolled and proceeded to the baseline/randomization visit (mean age 38.83 ± 9.91 years; range 23–69).

### 2.15 Randomization and study procedures

Eligible participants were randomized 1:1 to receive either Myoki + arginine + maltodextrin or arginine + maltodextrin (placebo, 40 per group) (Supplementary Table S1). The study was double-blind; participants, investigators, and study staff were unaware of treatment allocation throughout the trial. Participants were instructed to mix one sachet with 100 mL water and consume it after breakfast and a second sachet with 100 mL water after dinner for 12 weeks (two sachets/day; total daily peptide dose 200 mg in the Myoki arm).

Handgrip strength was assessed using a hand dynamometer and the 6-m walk test was performed according to the visit schedule at Screening (Visit 1), Randomization/Day 0 (Visit 2), Week 4 (Visit 3; Day 28 ± 4), Week 8 (Visit 4; Day 56 ± 4), and Week 12 (Visit 5; Day 84 ± 3). Muscle mass was evaluated using dual-energy X-ray absorptiometry (DEXA) at Screening/Baseline (Visit 1) and at Week 12 (Visit 5), and the 12-week change from baseline was used for efficacy analyses. Efficacy endpoints were evaluated as changes from baseline to Week 12; efficacy analyses were conducted in the per-protocol population, and safety analyses included all randomized participants who received at least one dose of study product (Supplementary Figure S2). Of 80 randomized participants, 75 completed the study.

### 2.16 Sample size calculation

The sample size was determined based on the primary endpoint, defined as the between-group difference in the change in muscle mass assessed by dual-energy X-ray absorptiometry (DEXA) from baseline to Week 12. A total of 72 participants were required to achieve 80% power at a two-sided α = 0.05, assuming a mean difference (test–placebo) of 0.3 and a standard deviation of 0.5. To account for an anticipated 10% dropout rate, 80 participants (40 per group) were planned to be randomized in a 1:1 allocation ratio.

### 2.17 Statistical analysis

#### 2.17.1 Preclinical experiments

All data were expressed as mean ± standard deviation (SD). Statistical analyses were performed using GraphPad Prism 10 (GraphPad Software, San Diego, CA, USA). For comparisons among more than two groups, one-way analysis of variance (ANOVA) was used, followed by Tukey’s multiple comparison test. Statistical differences were considered significant at p < 0.05 and were indicated as follows: *p < 0.05; **p < 0.01; ***p < 0.001.

#### 2.17.2 Clinical study

Statistical analyses were performed using SAS (version 9.4; SAS Institute Inc., Cary, NC, USA). Continuous variables were summarized as N, mean (SD), median, minimum, and maximum, and categorical variables as n (%), with percentages calculated as n/N × 100. Changes from baseline to Week 12 in DEXA-assessed muscle mass, 6-m walk test, and handgrip strength were analyzed using ANCOVA, adjusting for the corresponding baseline value. Least-squares means (LSMs), 95% confidence intervals, and two-sided p-values were reported, with p < 0.05 considered statistically significant.

## 3 Results

### 3.1 Morphological confirmation of Myoki-related cytotoxicity and myotube differentiation in C2C12 skeletal muscle cells

**FIGURE 1:**
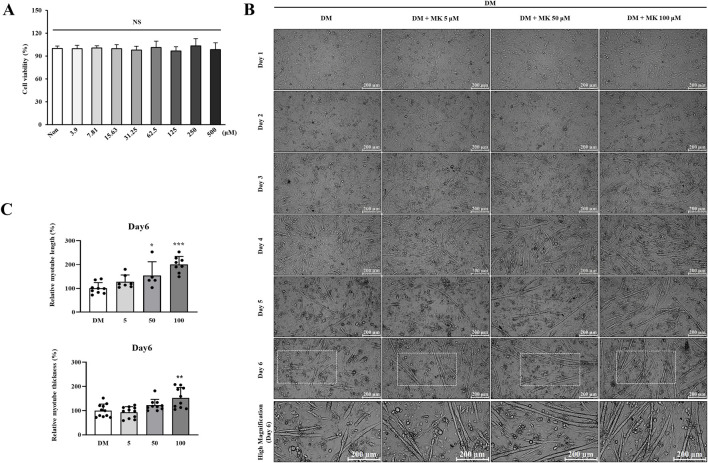
Myoki does not exhibit cytotoxicity and enhances myotube formation. **(A)** To assess the impact of Myoki on cell viability, C2C12 myoblasts were seeded and maintained throughout the experiment in growth medium (DMEM containing 10% FBS and 1% P/S) without differentiation induction. **(B)** To evaluate morphological changes following Myoki treatment during differentiation, C2C12 cells were cultured in differentiation medium (DM) with or without Myoki for 6 days, and phase-contrast images were acquired at ×100 magnification using light microscopy. Scale bars: 200 μm. **(C)** Quantification of Day 6 myotube morphology, including relative myotube length and relative myotube thickness. Data are expressed as means ± SD. Statistical analyses were performed using one-way ANOVA followed by Tukey’s multiple comparisons test. NS, not significant vs. Non group; *p < 0.05, **p < 0.01, ***p < 0.001 vs. DM group.

First, we used C2C12 cells to evaluate the potential cytotoxicity of Myoki. We seeded the cells on 96-well plates and incubated them for 24 h, then supplemented the cultures with Myoki at concentrations ranging from 0 to 500 μM and incubated them again for 24 h. Next, we assessed cell viability using a cell viability assay kit, and observed negligible reduction in cell viability at concentrations up to 500 μM, indicating that Myoki does not exhibit cytotoxicity at concentrations below 500 μM (Figure 1A).

Following myoblast proliferation, the C2C12 cells underwent serum starvation (2% horse serum) resulting in myoblast differentiation and fusing into myotubes. Therefore, to assess how Myoki affects muscle differentiation, we evaluated its ability to induce myotube formation using the same method as for the myotube formation induction assay. After treating the cells with serum, so ensure Myoki concentrations of 1, 10, and 100 μM, we incubated the cultures for 6 days, and captured cell images using an optical microscope on incubation Days 0, 1, 2, 3, 4, 5, and 6 (Figure 1B). In the Myoki treated groups, myotube length increased concentration- and time-dependently. On Day 6, the 100 μM Myoki-treated group exhibited a statistically significant increase in myotube length by 200% ± 33.88% compared to the control.

In summary, Myoki did not exhibit any cytotoxicity up to 500 μM and dose- and time-dependently promoted myotube formation in C2C12 cells.

### 3.2 Increased differentiation marker and myofibrillar component proteins in Myoki-treated C2C12 cells

**FIGURE 2:**
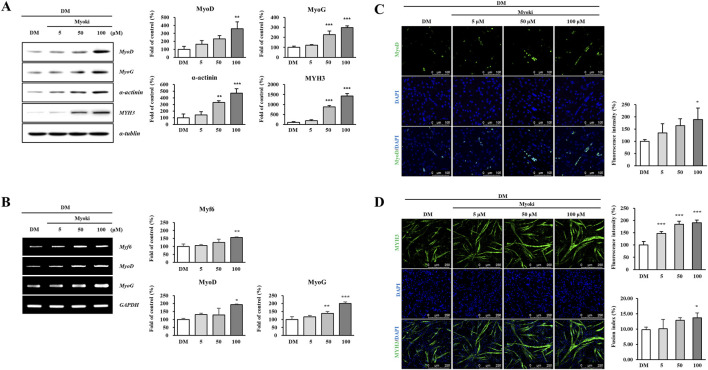
Myoki increases the expression of myogenic regulatory factors in C2C12 muscle cells. C2C12 myoblasts were cultured in differentiation medium (DM) with or without Myoki for 3 days. **(A)** Protein expression levels of MyoD, MyoG, α-actinin, and myosin heavy chain 3 (MYH3) were assessed by Western blotting. **(B)** mRNA levels of Myf6, MyoD, and MyoG were evaluated by RT-qPCR, and visualized by agarose gel electrophoresis. Immu-nofluorescence staining was performed to detect **(C)** MyoD and **(D)** MYH3 expression. Fluorescence intensity was quantified using ImageJ software. Scale bars: 100 μm (MyoD), 250 μm (MYH3). Data are expressed as means ± SD. Statistical analyses were performed using one-way ANOVA followed by Tukey’s multiple comparisons test. *p < 0.05, **p < 0.01, ***p < 0.001 vs. DM group.

To evaluate how Myoki affects the induction of C2C12 cell differentiation into myotubes, we cultured the cells in Myoki-supplemented differentiation medium. On Day 3 after Myoki treatment, Western blot analysis confirmed a significant increase in the expression of differentiation marker proteins, including myogenic regulatory factors (MRFs) such as MyoD and MyoG, as well as differentiated muscle fiber structural proteins like alpha-actinin and MYH3 (Figure 2A). In addition, RT-PCR analysis of mRNA isolated on Day 3 revealed a significant increase in MRF gene expression (Figure 2B). On Day 3 of the myotube differentiation stage, we stained MyoD and MYH3 with fluorescent antibodies and imaged the samples using a fluorescence microscope (Figures 2C,D). Our quantitative analysis revealed that treatment with 100 μM Myoki significantly increased MyoD and MYH3 expressions by 190% ± 46.28% and 191% ± 10.66%, respectively, compared to the control. In addition, the fluorescence-based fusion index increased from 9.80% ± 0.85% in the control group to 11.60% ± 3.32%, 12.86% ± 0.88%, and 13.66% ± 1.62% in the Myoki-treated groups (Figure 2D), supporting enhanced myotube multinucleation.

**FIGURE 3:**
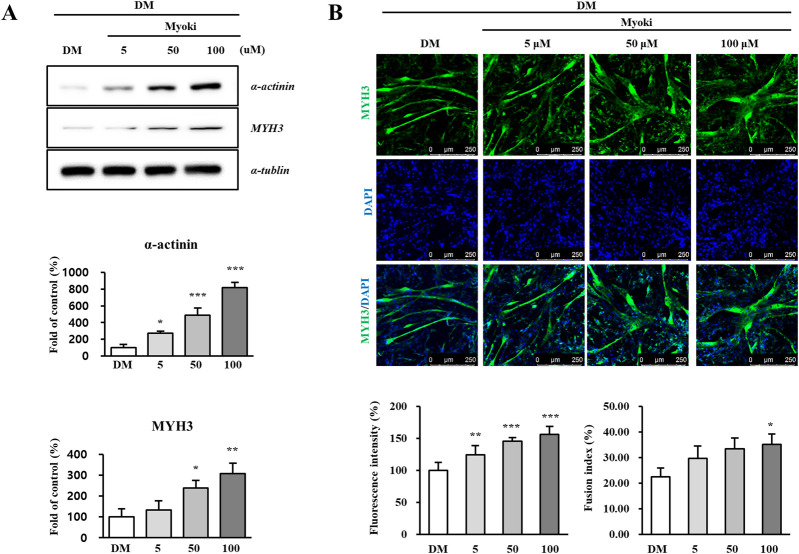
Myoki enhances the expression of key proteins that constitute the myofiber. C2C12 cells were cultured in differentiation medium (DM) with or without Myoki (5, 50, and 100 μM) for 6 days. **(A)** The expression levels of α-actinin and myosin heavy chain 3 (MYH3) were assessed by Western blotting. **(B)** MYH3 expression was further evaluated by immunofluorescence staining under the same conditions. Fluorescence intensity was quantified using ImageJ software. Scale bars: 250 μm. Data are expressed as means ± SD. Statistical analyses were performed using one-way ANOVA followed by Tukey’s multiple comparisons test. *p < 0.05, **p < 0.01, ***p < 0.001 vs. DM group.

On Day 6, Western blot analysis of the 100 μM Myoki treated group showed that the expression levels of alpha-actinin and MYH3 significantly increased by 818% and 307%, respectively (Figure 3A). Fluorescence staining of MYH3 on Day 6 also confirmed a significant increase of 153% compared to the control (Figure 3B). Consistently, the Day 6 fusion index increased from 22.54% ± 3.42% in the control group to 29.63% ± 4.87%, 33.41% ± 4.32%, and 35.16% ± 3.97% in the Myoki-treated groups (Figure 3B).

These results indicate that Myoki treatment significantly enhances differentiation marker and myofibrillar structural protein expressions during C2C12 myotube differentiation and is accompanied by an increase in fusion index, supporting improved myotube fusion.

### 3.3 Myoki attenuates DEX-Induced myotube atrophy through myostatin binding

**FIGURE 4:**
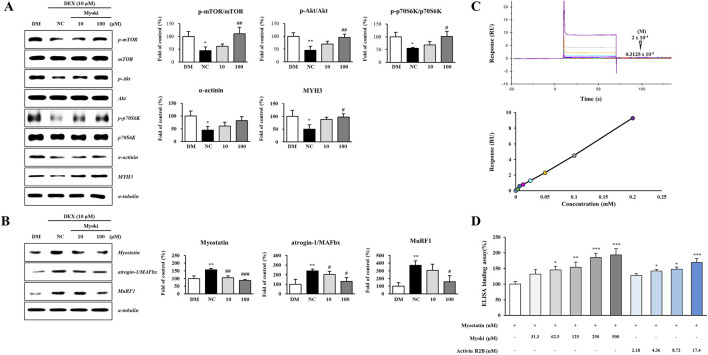
Myoki enhances protein synthesis and inhibits degradation in a DEX-induced C2C12 muscle atrophy model; SPR/ELISA assays support an *in vitro* interaction between Myoki and myostatin. C2C12 cells were differentiated for 5 days to form myotubes (DM group: differentiation medium only, no DEX or Myoki) and then treated with dexamethasone (DEX) to induce muscle atrophy, followed by culture with Myoki (10, 100 μM). **(A)** The expression levels of protein synthesis-related proteins and muscle fiber structural proteins, and **(B)** protein degradation-related proteins were analyzed by Western blotting. **(C)** SPR analysis revealed concentration-dependent binding of the peptide to the myostatin-immobilized chip (3.125–200 μM; from bottom to top), and the equilibrium responses were fitted to a steady-state affinity model. **(D)** An ELISA-based binding assay was conducted to assess the interaction between Myoki and myostatin. Data are expressed as means ± SD. Statistical analyses were performed using one-way ANOVA followed by Tukey’s multiple comparisons test. *p < 0.05, **p < 0.01, ***p < 0.001 vs. DM (or myostatin-only) group. ^#^ *p* < 0.05, ^##^ *p* < 0.01, ^###^ *p* < 0.001 vs. negative control (NC) group.

Next, we established an *in vitro* muscle atrophy model by exposing C2C12 myotubes to 10 μM DEX. Myoki treatment effectively attenuated the DEX-induced damage in C2C12 cells. The DEX treatment significantly reduced the phosphorylation levels of the muscle growth signaling proteins and the expression levels of differentiated muscle fiber structural proteins, which were dose-dependently restored toward control levels in the Myoki treatment groups (Figure 4A). We performed a Western blot analysis to assess the expression levels of atrogin-1/MAFbx and MuRF1, representing essential proteins for activating the ubiquitin-proteasome system of muscle protein degradation. We observed a significant increase in the DEX-treated group compared to the control. However, in the Myoki treatment groups, their expression levels were significantly and dose-dependently reduced (Figure 4B). These findings suggest that Myoki might block the DEX-induced myotube atrophy pathway and prevent muscle atrophy. Based on this result, we further investigated the underlying mechanism of the myostatin inhibitory activity of Myoki by confirming its binding to myostatin. To determine the binding affinity between the Myoki peptide complex and human myostatin, we performed SPR analysis using the Biacore T200 system (GE Healthcare) and verified that the peptide binds to Myostatin. We injected seven different peptide concentrations, ranging from 3.125 μM to 200 μM, over a CM5 chip immobilized with Myostatin-Fc, and SPR obtained sensorgrams. Although we observed relatively rapid association and dissociation, the RUs increased dose-dependently (Figure 4C). Moreover, we performed a receptor binding assay using ELISA and demonstrated that Myoki directly and concentration-dependently binds to myostatin. We coated Myoki at concentrations of 125, 250, 500, and 1,000 μM and the myostatin receptor activin receptor 2B (activin R2B) at concentrations of 2.18, 4.36, 8.72, and 17.44 nM on ELISA plates. As we tested activin R2B in the nanomolar range (being approximately 10^3-fold lower than the micromolar range of Myoki), Figure 4D should be interpreted with caution and not as a direct affinity comparison. Upon myostatin treatment, Myoki significantly enhanced myostatin binding by more than 150% compared to the control, which was observed at concentrations ≥62.5 μM (Figure 4D).

In summary, Myoki effectively attenuated DEX-induced myotube atrophy by dose-dependently restoring protein synthesis-related signaling and structural proteins, while binding and inhibiting myostatin.

### 3.4 Myoki promotes recovery from muscle atrophy in an accelerated aging animal model

**FIGURE 5:**
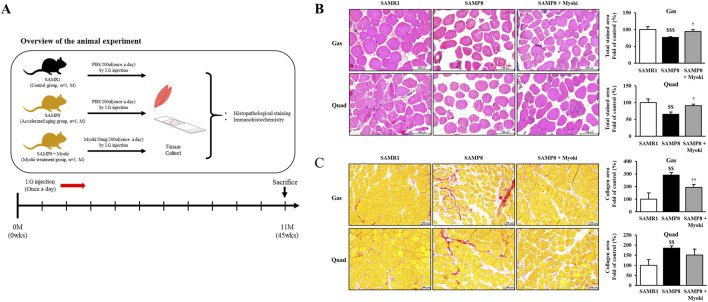
Histological analysis shows that Myoki treatment alleviates muscle damage and fibrosis in senescence-accelerated mice (SAMP8). **(A)** SAMP8 mice were treated with Myoki peptide *via* intragastric (I.G.) administration for 45 weeks. Gastrocnemius and quadriceps muscles were collected, fixed in 10% neutral-buffered formalin, embedded in paraffin, sectioned, and stained with **(B)** hematoxylin and eosin (H&E) for general histological assessment and **(C)** Sirius Red for collagen fiber detection. Histological features were evaluated by light microscopy. Scale bars: 100 μm. Data are expressed as means ± SD. Statistical analyses were performed using one-way ANOVA followed by Tukey’s multiple comparisons test. ^$$^ *p* < 0.01, ^$$$^ *p* < 0.001 vs. SAMR1. ^**†**^ *p* < 0.05, ^**††**^ *p* < 0.01 vs. SAMP8 group.

Next, we designed an experiment using the accelerated aging mouse model SAMP8 (Figure 5A). We performed H&E staining of the gastrocnemius (Gas) and quadriceps (Quad) muscles to assess myofiber morphology and cross-sectional area. Compared with the SAMR1 group, the SAMP8 group displayed a significant myofiber area reduction both muscles, while Myoki treatment significantly increased the myofiber area in both muscles compared to the SAMP8 group (Figure 5B).

We applied Sirius Red staining to evaluate collagen deposition (fibrotic remodeling) in Gas and Quad. As Picrosirius Red highlights fibrillar collagen, it is widely used to quantitatively assess interstitial collagen accumulation, and collagen-positive area was quantified under standardized conditions. Importantly, in skeletal muscle, increased interstitial collagen/ECM deposition is commonly interpreted as fibrotic remodeling, which can increase tissue stiffness and impair effective force transmission as well as the regenerative microenvironment, including muscle stem/progenitor cell activity (Csapo et al., 2020). Collagen accumulation was markedly increased in the SAMP8 group compared with SAMR1. In the Myoki-treated group, collagen deposition in Gas decreased from 291% ± 19.72% to 194% ± 22.84% (normalized to SAMR1 = 100%). In Quad, collagen deposition decreased from 185% ± 10.61% to 151% ± 28.82%, although this difference did not reach the level of statistical significance (Figure 5C).

Overall, Myoki treatment improved muscle fiber area in the SAMP8 mouse model, suggesting its potential to mitigate muscle aging.

### 3.5 Myoki alters image-based fluorescence intensity signals related to muscle atrophy and muscle fiber type markers in SAMP8 mice

**FIGURE 6:**
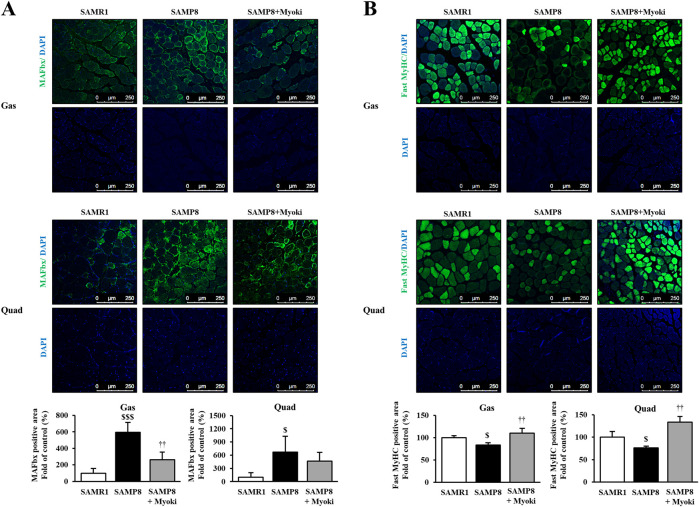
Myoki peptide counteracts muscle atrophy-associated changes in the skeletal muscles of senescence-accelerated mice (SAMP8). Gastrocnemius and quadriceps muscles from SAMP8 mice treated with or without Myoki were collected, embedded in paraffin, and sectioned for immunofluorescence analysis. Sections were stained with antibodies against **(A)** atrogin-1/MAFbx, and **(B)** fast myosin heavy chain (MyHC) (green) to evaluate the expression of key proteins involved in muscle atrophy and fiber composition. Nuclei were counterstained with DAPI (blue). Representative images were captured by fluorescence microscopy, and fluorescence intensity was quantified using ImageJ software. Scale bars: 250 μm. Data are expressed as means ± SD. Statistical analyses were performed using one-way ANOVA followed by Tukey’s multiple comparisons test. ^$^ *p* < 0.05, ^$$$^ *p* < 0.001 vs. SAMR1. ^**††**^ *p* < 0.01 vs. SAMP8 group.

We examined the effects of Myoki on muscle atrophy–related phenotypes in the senescence-accelerated mouse model SAMP8 (Figure 5A). We fluorescently stained the Gas and Quad muscles harvested from mice to evaluate image-based changes in the fluorescence intensity of atrogin-1/MAFbx, an atrophy-related ubiquitin–proteasome system (UPS) marker (i.e., a signal related to muscle atrophy), under standardized staining and imaging conditions. Our results indicated that the fluorescence intensities of atrogin-1/MAFbx significantly increased both in the Gas and Quad muscles of the SAMP8 group compared to the SAMR1 group. Notably, in the Myoki-treated group, fluorescence intensities significantly decreased in the Gas muscle compared to the SAMP8 group (Figure 6A). Furthermore, fluorescence intensity of fast myosin heavy chain (fast-MyHC), a major contractile protein and a marker of fast-twitch muscle fibers in the skeletal muscle, decreased in the SAMP8 group, but was restored toward SAMR1 levels upon the Myoki treatment (Figure 6B).

Collectively, Myoki treatment decreased atrogin-1/MAFbx fluorescence intensity in Gas and Quad muscles of SAMP8 mice and increased fast-MyHC fluorescence intensity toward SAMR1 levels.

### 3.6 Randomized, double-blind, placebo-controlled, parallel study to evaluate the efficacy and safety of Myoki use in patients with muscle atrophy

**FIGURE 7:**
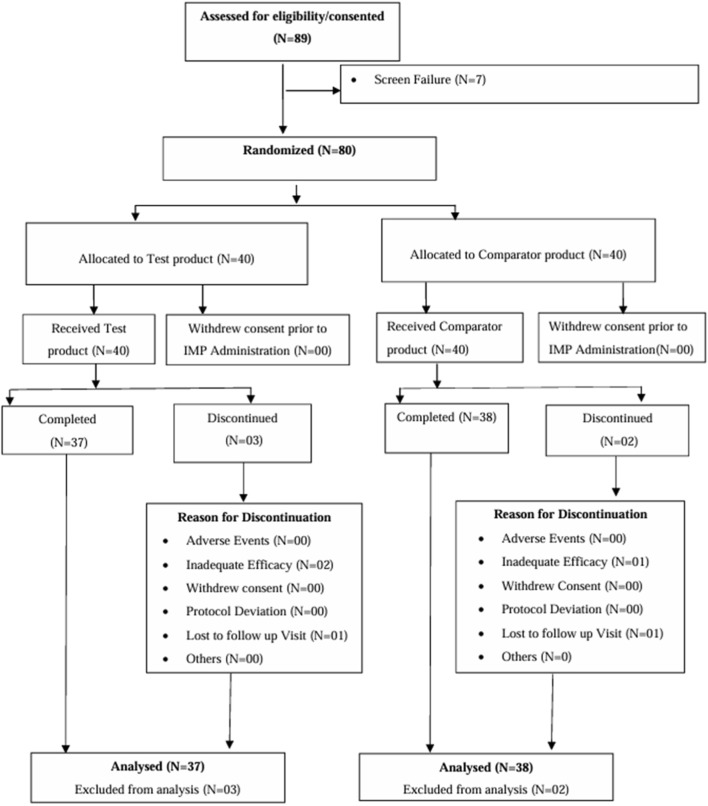
Details of disposition of patients in the study.

To evaluate the clinical efficacy and safety of Myoki application, we conducted a 12-week randomized, double-blind, placebo-controlled, parallel study in adults with muscle atrophy. The enrolled cohort retained a mean age of 38.83 ± 9.91 years (range: 23–69 years), indicating that the participants were not restricted to older adults and thus represented an adult population with muscle atrophy. Participants met prespecified criteria for low muscle mass by DEXA (men <7.0 kg/m^2^; women <5.4 kg/m^2^) along with impaired muscle function, defined by low handgrip strength and reduced physical performance (6-m walk speed <1.0 m/s). The per-protocol efficacy analyses included 75 participants (placebo, *n* = 37; Myoki, *n* = 38). Participant flow through the study is shown in Figure 7. Table 1 summarizes the baseline demographic and functional characteristics (including mobility measures).

**TABLE 1:** Baseline demographic and other clinical characteristics.

Demographic and clinical characteristic	Placebo (*N* = 40)	Treatment (*N* = 40)	*p*-value
	Mean ± SD	Mean ± SD	
Age (in completed years), mean (SD) [range]	38.65 ± 8.55 [23–69]	39.00 ± 11.22 [23–63]	​
Gender *n* (%)	​	​	0.8757
Female *n* (%)	10 (25.00%)	9 (22.50%)	​
Male *n* (%)	30 (75.00%)	31 (77.50%)	​
Ethnicity *n* (%)	​	​	​
Asian	40 (100.00%)	40 (100.0%)	​
Weight - kg	54.79 ± 9.41	56.17 ± 10.05	0.5270
Height - cm	163.00 ± 7.61	164.34 ± 8.29	0.4514
BMI - kg/m^2^	20.59 ± 3.02	20.70 ± 2.78	0.8568
Muscle mass - kg/m^2^	5.84 ± 0.78	5.96 ± 0.71	​
6-meter walk test - seconds	8.99 ± 1.82	9.30 ± 2.07	​
Hand grip strength test - kg	​	​	​
Left hand - kg	19.59 ± 4.31	20.40 ± 3.83	​
Right hand - kg	20.49 ± 4.19	20.63 ± 4.40	​

**FIGURE 8:**
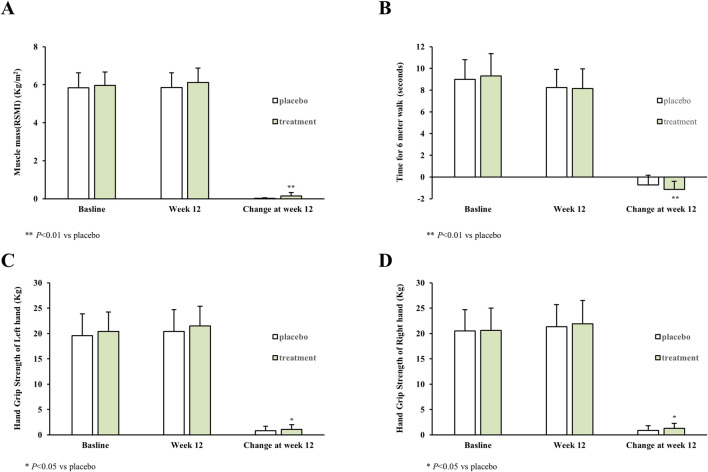
Changes in muscle mass and physical performance from baseline to Week 12 (Myoki vs placebo) **(A)** Muscle mass index (RSMI, kg/m^2^) assessed by dual-energy X-ray absorptiometry (DEXA). **(B)** Time to complete the 6-m walk test (seconds). **(C)** Left-hand grip strength (kg). **(D)** Right-hand grip strength (kg). Bars represent mean ± SD at baseline and Week 12, and the change from baseline to Week 12 (Week 12 − baseline). Data are shown for the per-protocol set (placebo, *n* = 37; Myoki, *n* = 38). Quantitative changes, adjusted between-group differences (95% CI), and *p*-values are provided in Table 2.

From the baseline to Week 12, DEXA-assessed lean mass increased more in the Myoki than in the placebo group (LS mean change: 930.8 g vs 225 g), with an adjusted between-group difference of 705.8 g (95% CI, 433.40 to 978.16; *p* < 0.01) (Table 2). Similarly, the change in muscle mass index favored Myoki (LS mean change: 0.15 vs 0.02 kg/m^2^), with an adjusted difference of 0.13 kg/m^2^ (95% CI, 0.07 to 0.19; *p* < 0.01) (Table 2). Consistent with these findings, Figure 8A illustrates the baseline and Week 12 values and the corresponding change from baseline for muscle mass index in both groups. Functional performance assessed by time to complete the 6-m walk test improved in both groups, with a more marked improvement in the Myoki group (LS mean change: −1.12 s vs −0.77 s; between-group difference −0.34 s; 95% CI, −0.68 to 0.00; *p* < 0.01) (Table 2). This improvement is also depicted in Figure 8B. Handgrip strength increased in both groups, with between-group differences of 0.27 and 0.43 kg for the left and right hands (95% CI, −0.15 to –0.69 and −0.04 to –0.89; *p* = 0.02 and 0.04), respectively (Table 2). Changes in left- and right-hand grip strength are shown in Figures 8C,D, respectively, consistent with Table 2.

**TABLE 2:** Week 12 changes from baseline in muscle mass, physical performance, and serum biomarkers.

End point	Placebo (*N* = 37)	Treatment (*N* = 38)	Difference between placebo and treatment (95% CI)	*p*-value
Lean muscle mass (g)	225	930.8	705.8 (433.40 to 978.16)	<0.01**
95% CI for LS mean	(33.69 to 416.31)	(736.90 to 1124.7)		
Muscle mass (kg/m^2^)	0.02	0.15	0.13 (0.07 to 0.19)	<0.01**
95% CI for LS mean	(−0.02 to 0.06)	(0.11 to 0.19)		
Time to complete 6-meter walk test (TTC-6mWT) (sec)	−0.77	−1.12	−0.34 (−0.68 to 0.00)	<0.01**
95% CI for LS mean	(−1.01 to −0.53)	(−1.36 to −0.87)		
Hand grip strength test (Left hand) (kg)	0.81	1.07	0.27 (−0.15 to 0.69)	0.02*
95% CI for LS mean	(0.51 to 1.10)	(0.77 to 1.37)		
Hand grip strength test (Right Hand)(kg)	0.85	1.27	0.43 (−0.04 to 0.89)	0.04*
95% CI for LS mean	(0.52 to 1.17)	(0.94 to 1.60)		
TNF-alpha (pg/mL)	4.3	3.97	−0.33 (−8.07 to 7.42)	0.933
95% CI for LS mean	(−1.14 to 9.74)	(−1.54 to 9.48)		
IGF-1 (ng/mL)	13.51	31.44	17.93 (1.00 to 34.87)	0.038*
95% CI for LS mean	(1.71 to 25.30)	(19.49 to 43.40)		
Sex hormones (Estrogen) (pg/mL)	−1.98	−2.37	−0.39 (−8.45 to 7.67)	0.923
95% CI for LS mean	(−7.64 to 3.68)	(−8.11 to 3.36)		
Sex hormones (testosterone) (pg/mL)	−29.9	46.54	76.44 (−18.68 to 171.56)	0.114
95% CI for LS mean	(−96.63 to 36.83)	(−21.09 to 114.17)		
IL-6 (pg/mL)	1.31	0.04	−1.27 (−4.97 to 2.43)	0.496
95% CI for LS mean	(−1.28 to 3.90)	(−2.59 to 2.67)		
Myoglobin (ng/mL)	8.15	−6.45	−14.6 (−20.06 to −9.14)	<0.01**
95% CI for LS mean	(4.32 to 11.98)	(−10.33 to −2.56)		
Creatine kinase (CK-MB) (ng/mL)	3.41	−0.54	−3.95 (−6.18 to −1.72)	<0.01**
95% CI for LS mean	(1.84 to 4.97)	(−2.13 to 1.04)		
Aspartate aminotransferase	2.57	−2.83	−5.39 (−10.09 to −0.70)	0.025*
95% CI for LS mean (IU/L)	(−0.70 to 5.84)	(−6.14 to 0.49)		

*p < 0.05, **p < 0.01.

Among the biomarkers, TNF-α, estrogen, testosterone, and IL-6 did not differ between the groups (all *p* > 0.05). In contrast, IGF-1 increased more in the Myoki than in the placebo group (between-group difference 17.93 ng/mL; 95% CI, 1.00 to 34.87; *p* = 0.038), while myoglobin, CK-MB, and aspartate aminotransferase decreased more in the Myoki group (myoglobin: −14.6 ng/mL [95% CI, −20.06 to −9.14], *p* < 0.01; CK-MB: −3.95 ng/mL [95% CI, −6.18 to −1.72], *p* < 0.01; AST: −5.39 IU/L [95% CI, −10.09 to −0.70], *p* = 0.025) (Table 2). Safety was assessed in all randomized participants (*n* = 80; 40 per group); Treatment Emergent Adverse Event (TEAEs) occurred in 10/40 (25.0%) participants in the Myoki group (10 events) and 7/40 (17.5%) participants in the placebo group (seven events) (Supplementary Table S2). No TEAEs were assessed as related to the study product, no serious TEAEs were reported, and all TEAEs were mild in severity and resolved.

## 4 Discussion

In this study, we investigated how the synthetic peptide Myoki affects muscle formation and atrophy. Our results from the *in vitro* assays, accelerated aging mouse model, and clinical trial in patients with muscle atrophy collectively suggest that Myoki might attenuate muscle atrophy-associated phenotypes and it binds myostatin *in vitro*. However, in this study, we did not establish a causal mechanistic link between this interaction and the observed anti-atrophic effects. Accordingly, we have reframed myostatin binding as a supportive evidence and working hypothesis rather than a definitive mechanism. These results are in good agreement with prior findings demonstrating that myostatin inhibition induced muscle hypertrophy and prevented atrophy in various models (Lee and McPherron, 2001; Amthor et al., 2009). Furthermore, clinical outcomes revealed that Myoki significantly improved muscle strength and physical function in patients with muscle atrophy compared to placebo. However, such clinical outcomes could not be directly attributed to Myoki–myostatin binding based on the current evidence, and should be interpreted independently of the *in vitro* binding data pending future mechanistic validation. These improvements are consistent with the results of previous studies describing that myostatin pathway targeting could enhance muscle performance in older adult or atrophic populations (Rooks and Roubenoff, 2019; Lee et al., 2023). These results provide experimental evidence supporting the therapeutic potential of Myoki in muscle-related disorders, suggesting its promising potential in muscle atrophy and sarcopenia treatment.

First, Myoki was confirmed to be non-cytotoxic even at a concentration of 500 μM. Notably, at 100 μM (i.e., one-fifth the concentration of the non-toxic threshold) Myoki significantly increased myotube length and upregulated muscle differentiation marker proteins (e.g., MyoD and MyoG) as well as structural muscle proteins, including MYH3 and alpha-actinin. Similar effects have been observed in the case of vitamin D and creatine, both of which are well-known for muscle synthesis and strength enhancement (Braga et al., 2017; Deldicque et al., 2007). These substances support myogenic differentiation by modulating MRFs, including MyoD and MyoG. These results suggest that Myoki could effectively promote myogenic differentiation and facilitate muscle fiber formation in C2C12 cells, even at low doses.

Another important finding is the capacity of Myoki to suppress muscle atrophy. Myostatin induces atrogin-1/MAFbx and MuRF1 expression, thereby promoting proteasome-mediated protein degradation (Durieux et al., 2007). SPR- and ELISA-based binding assays confirmed that Myoki binds myostatin, an interaction suggesting that Myoki might modulate myostatin-driven activation of the ubiquitin–proteasome system, thereby reducing excessive protein breakdown and muscle loss. In addition, the Akt/mTOR signaling pathway is central to promoting skeletal muscle hypertrophy and counteracting muscle atrophy (Bodine et al., 2001b; Kitakaze et al., 2016). Myoki restored the phosphorylation of key signaling proteins (e.g., Akt, mTOR, and p70S6K), consistent with enhanced protein synthesis. Therefore, Myoki might retain the potential to enhance muscle protein synthesis while simultaneously suppressing protein degradation pathways.

The SAMP8 mouse model is widely used in aging research and has been applied to muscular aging/sarcopenia studies as it exhibits increased oxidative stress and inflammation and develops age-associated skeletal muscle atrophy (Bayram et al., 2013; Yun et al., 2015; Morita et al., 2021). In this aging model, Myoki significantly increased muscle fiber area and effectively improved fibrotic muscle tissue. In addition, the recovery of fast-MyHC expression suggested the partial restoration of contractile protein composition, supporting structural recovery. In our randomized, double-blind, placebo-controlled clinical study involving muscle atrophy patients, participants who received Myoki displayed significant improvements in muscle mass, hand grip strength, and walking speed. IGF-I levels, representing a critical element for muscle growth (Yoshida and Delafontaine, 2020), also increased. In addition, Myoki reduced myoglobin and creatine kinase levels that reportedly increase during excessive muscle damage (Baird et al., 2012; Driessen-Kletter et al., 1990), suggesting its potential role in muscle recovery. Furthermore, arginine was included in the treatment strategy as a supportive amino acid component, and clinical studies of arginine-containing anabolic formulations in older adults described improved protein metabolism and lean tissue along with muscle strength and physical performance gain (Flakoll et al., 2004; Baier et al., 2009; Børsheim et al., 2008). In addition, reduced arginine availability has been reported in aging and sarcopenic phenotypes, supporting its inclusion as a supportive nutritional component in muscle-wasting settings (Hua et al., 2024). As we included arginine both in the Myoki and placebo formulations, the between-group improvements primarily reflect the additional Myoki effect beyond this shared background component. However, we did not assess myostatin-pathway biomarkers and target engagement in trial participants, precluding the mechanistic attribution of the clinical outcomes to Myoki–myostatin binding. In summary, Myoki treatment could help attenuate muscle atrophy progression.

Despite the encouraging results, several important limitations should be considered when interpreting the discoveries of this study. First, in the SAMP8 accelerated aging model, we primarily relied on histological (H&E and Sirius Red) and protein-level immunofluorescence (atrogin-1/MAFbx, and fast-MyHC) analyses, and did not perform parallel mRNA expression profiling or direct physiological assessments such as grip strength or treadmill/endurance testing in these animals. While these data support improvements at the histological and protein marker levels, additional transcript-level analyses and functional measurements in aged animal models would be important to further strengthen the *in vivo* results obtained for Myoki. Second, the incomplete understanding of the molecular mechanisms through which Myoki exerts its effects represents a major concern. Although myostatin inhibition appears to be the underlying mechanism, we did not directly and sufficiently assess myostatin pathway activation or target engagement to establish causality *in vivo*. Muscle homeostasis is regulated by a complex signaling network, and whether Myoki influences alternative pathways or triggers compensatory responses remains unclear. Moreover, as we did not include arginine in the C2C12 experiments, our *in vitro* data reflect Myoki-only effects without addressing whether arginine modulates muscle protein signaling, which should be tested directly in future mechanistic studies. A further limitation concerns the clinical trial component, which, despite the promising outcomes, was limited both in scope and duration. We based the observed muscle mass-, strength-, and function-related improvement on a relatively small participant pool with a short follow-up period. Consequently, whether these benefits are sustainable over the long term remains uncertain. Moreover, although we performed this study in participants with muscle atrophy, the mean age of the participants was relatively low compared to typical sarcopenia cohorts, limiting the generalizability of our results to older populations. Accordingly, dedicated trials in older adults with sarcopenia would be required to directly confirm efficacy in that specific population. Finally, we did not evaluate Myoki along with standard therapeutic strategies, such as resistance training or nutritional supplementation, both widely recommended for managing muscle atrophy and sarcopenia. Therefore, whether Myoki offers additional benefits beyond conventional treatments or produces synergistic effects when used in combination remain to be elucidated.

## 5 Conclusion

In summary, we demonstrated that Myoki, a synthetic peptide, promotes myogenesis and suppresses muscle atrophy in C2C12 myoblasts, and suggest myostatin binding as a possible underlying mechanism to this effect. Moreover, Myoki reduced aging-related muscle atrophy markers in the muscle tissues of an aged mouse model. Furthermore, in a clinical study involving patients with muscle atrophy, Myoki significantly improved muscle mass and strength, walking speed, and blood biomarkers. Therefore, both our preclinical and clinical studies demonstrated the consistently positive impact of Myoki in preventing muscle atrophy and underpinned its potential as a promising therapeutic agent against conditions such as sarcopenia and muscle atrophy.

## Data Availability

The original contributions presented in the study are included in the article/Supplementary Material, further inquiries can be directed to the corresponding author.

## Glossary

2CTC: 2-Chlorotrityl Chloride (resin)
Akt: Protein Kinase B
ANCOVA: Analysis of Covariance
ANOVA: Analysis of Variance
AST: Aspartate Aminotransferase
ATCC: American Type Culture Collection
BSA: Bovine Serum Albumin
C18: Octadecylsilane (C18)
CDSCO: Central Drugs Standard Control Organization
CK-MB: Creatine Kinase-MB
CM5: Carboxymethylated dextran sensor chip (Biacore CM5)
CTRI: Clinical Trials Registry-India
DAPI: 4′,6-diamidino-2-phenylindole
DCM: Dichloromethane
DEX: Dexamethasone
DEXA: Dual-Energy X-ray Absorptiometry
DIPEA: N,N-diisopropylethylamine
DM: Differentiation Medium
DMEM: Dulbecco’s Modified Eagle’s Medium
DMF: N,N-dimethylformamide
ECL: Enhanced Chemiluminescence
EDC: 1-Ethyl-3-(3-dimethylaminopropyl)carbodiimide
EDT: 1,2-ethanedithiol
ELISA: Enzyme-Linked Immunosorbent Assay
ESI: Electrospray Ionization
EtBr: Ethidium Bromide
FBS: Fetal Bovine Serum
Fmoc: 9-fluorenylmethoxycarbonyl
GAPDH: Glyceraldehyde 3-Phosphate Dehydrogenase
Gas: Gastrocnemius muscle
GCP: Good Clinical Practice
GDF-8: Growth Differentiation Factor-8
GM: Growth Medium
H&E: Hematoxylin and Eosin
HBS-EP: Hepes Buffered Saline with EDTA and P20
HBTU: 2-(1H-benzotriazol-1-yl)-1,1,3,3-tetramethyluronium hexafluorophosphate
HPLC: High-Performance Liquid Chromatography
HRP: Horseradish Peroxidase
ICD-10-CM: International Classification of Diseases, 10th Revision, Clinical Modification
ICH-GCP: International Council for Harmonisation–Good Clinical Practice
ICMR: Indian Council of Medical Research
I.G.: Intragastric
IGF-I: Insulin-like Growth Factor I
IgG: Immunoglobulin G
IF: Indirect immunofluorescence
IL-6: Interleukin-6
LC–MS/MS: Liquid Chromatography with Tandem Mass Spectrometry
LS: Least Squares
LSMs: Least-squares means
MAFbx: Muscle Atrophy F-box protein
MRFs: Myogenic Regulatory Factors
MS/MS: Tandem Mass Spectrometry
MSTN: Myostatin
mTOR: Mechanistic Target of Rapamycin
MTBE: Methyl tert-butyl ether
MuRF1: Muscle RING Finger-1
Myf5: Myogenic Factor 5
Myf6: Myogenic Factor 6
MyHC: Myosin Heavy Chain
MYH3: Myosin Heavy Chain 3
MyoD: Myoblast determination protein 1
NC: Negative Control
NHS: N-Hydroxysuccinimide
NIH: National Institutes of Health
NS: Not Significant
p70S6K: Ribosomal protein S6 kinase beta-1
PBS: Phosphate-Buffered Saline
PBST: Phosphate-buffered saline with Tween-20
P/S: Penicillin–Streptomycin
PVDF: Polyvinylidene Fluoride
PW: Purified Water
Quad: Quadriceps muscle
R2B: Activin Receptor Type-2B
RT-PCR: Reverse Transcription Polymerase Chain Reaction
RT-qPCR: Real Time quantitative Polymerase Chain Reaction
RUs: Response Units
SAS: Statistical Analysis System
SAMP8: Senescence-Accelerated Mouse Prone 8
SAMR1: Senescence-Accelerated Mouse Resistant 1
SDS-PAGE: Sodium Dodecyl Sulfate–Polyacrylamide Gel Electrophoresis
SD: Standard Deviation
SPR: Surface Plasmon Resonance
TFA: Trifluoroacetic Acid
TIS: Triisopropylsilane
TMB: 3,3′,5,5′-Tetramethylbenzotidine
TNF-α: Tumor Necrosis Factor-alpha
TRS: Target Retrieval Solution
TTC-6mWT: Time to Complete 6-meter Walk Test
RSMI: Relative Skeletal Muscle Index
TEAE: Treatment Emergent Adverse Event.
