# Supplementary material for: The therapeutic potential of Myoki, a novel peptide in muscle atrophy: mechanisms and applications
Source: Front Pharmacol. 2026 Feb 12;17:1663850. doi: 10.3389/fphar.2026.1663850 (PMC12935919; doi:10.3389/fphar.2026.1663850)
Supplement: Supplementary file 1 [file Supplementaryfile1.docx]

**
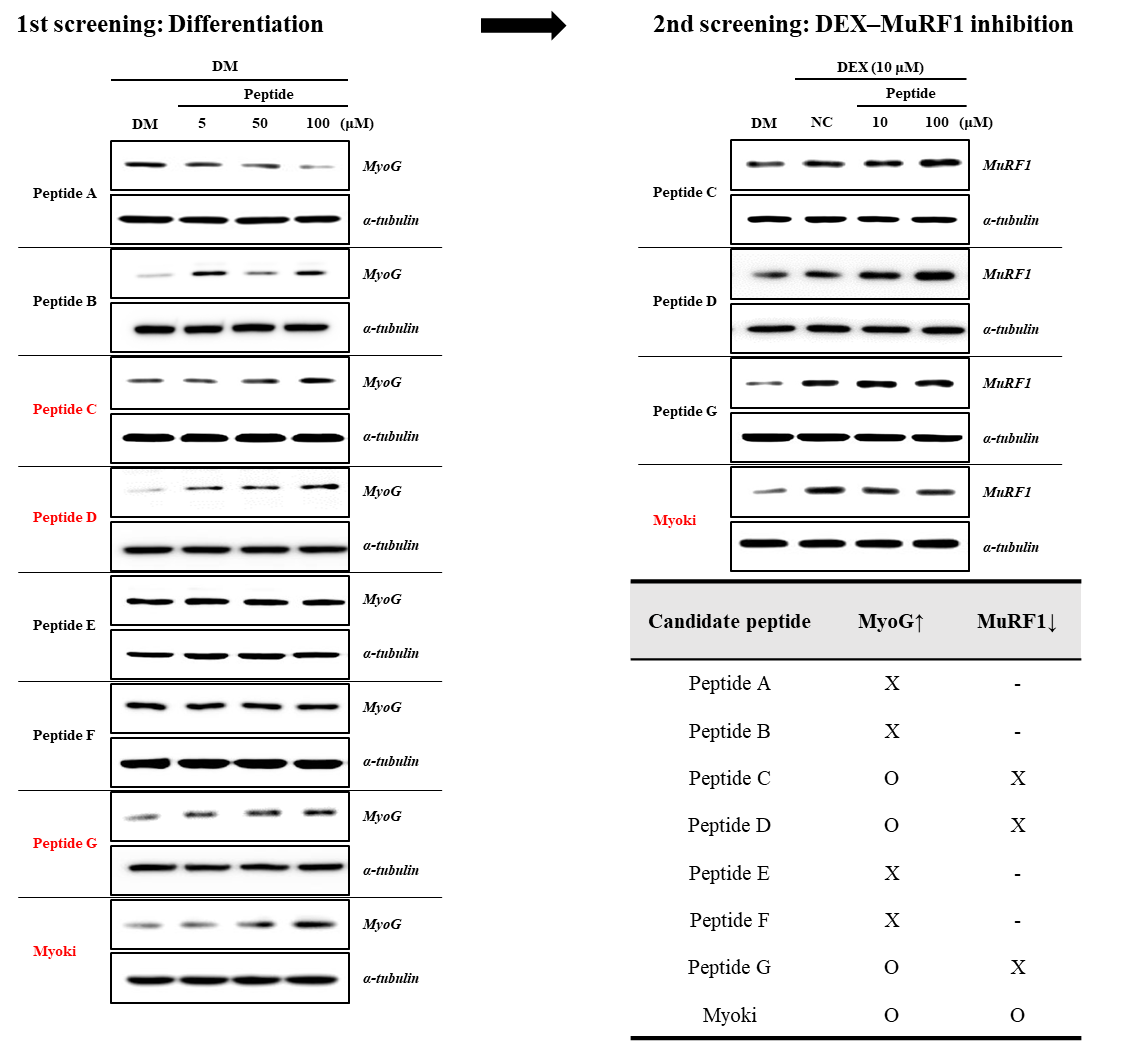
**

**Supplementary Figure 1.** Preliminary screening of candidate peptides identifies Myoki as a lead candidate in C2C12 cells. Candidate peptides (A–G) and Myoki were screened in C2C12 cells for pro-myogenic activity (increased MyoG expression during differentiation) and anti-catabolic activity (attenuation of DEX-induced MuRF1 upregulation). Western blot images from the screening experiment are shown, and the summary table indicates peptides that met each criterion (O, yes; X, no; –, not determined).

**
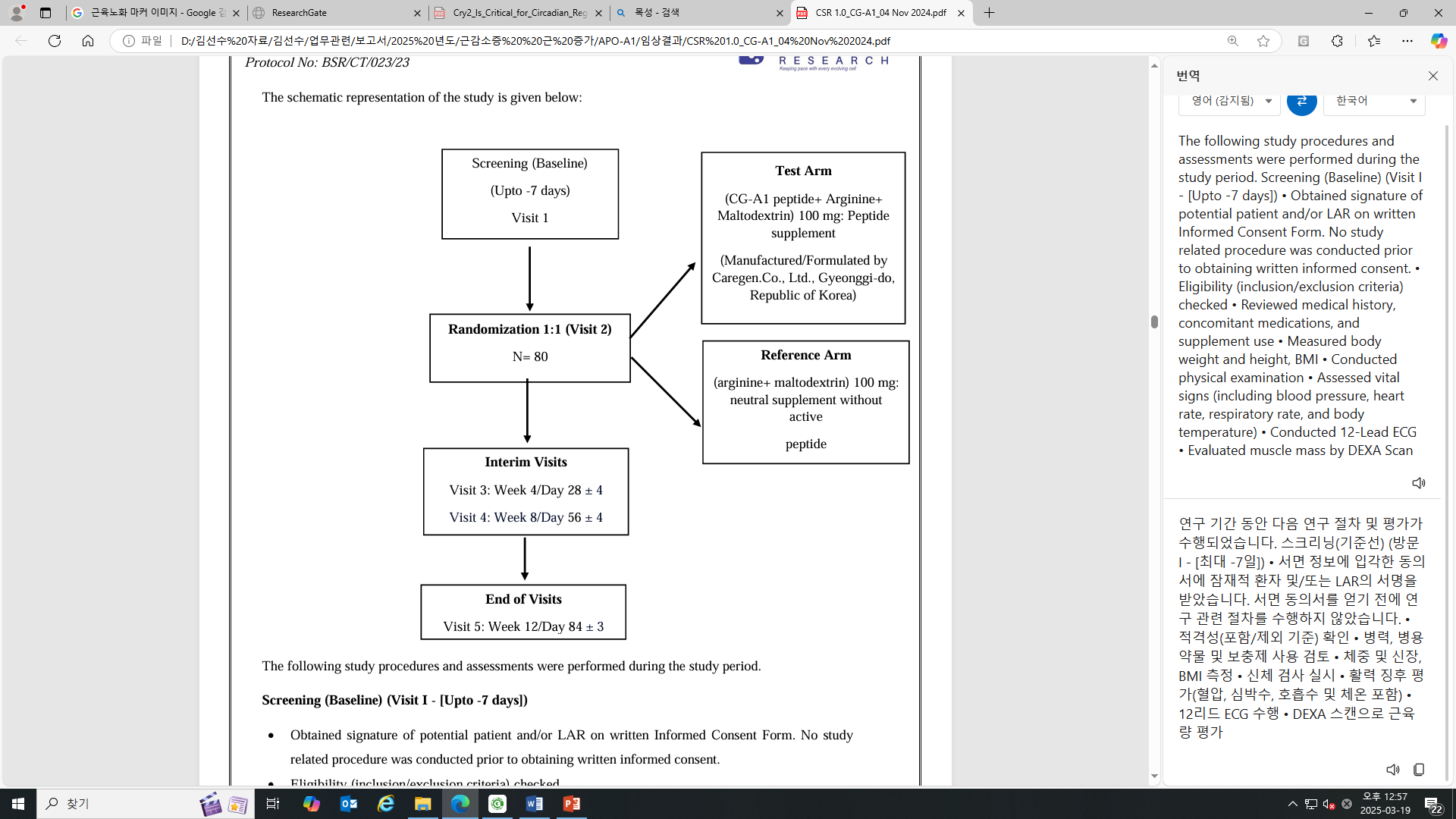
Supplementary Figure 2.** Schematic overview of the study

**Supplementary Table 1.** Inclusion and exclusion criteria

| **Inclusion criteria** | **Exclusion criteria** |
| --- | --- |
| - Male and female patients who are 18 years of age and older of any ethnicity. - Patients with pathologic or physiologic muscle atrophy   (muscle mass assessed by DEXA Scan) (Male: <7.0 kg/m2, Female: <5.4 kg/m2)   - Patients with low muscle strength   (defined as <28 kg for men and <18 kg for women assessed by hand dynamometer) or low physical performance assessed by 6-meter walk test (6-meter walk:<1m/s )   - Patients who are accustomed to normal levels of physical activity except for bed- ridden patients (low physical performance patients) - Body Mass Index (BMI) <25 kg/m2. - Non-smoker. - Vital signs are normal or deemed abnormal but clinically insignificant by the Investigator. - Clinical laboratory evaluations (clinical biochemistry, hematology) are normal or deemed clinically insignificant by the Investigator. - Female patients who are: - Post-menopausal (absence of menses for 1 year or more)**,** Surgically sterilized - WOCBP who are using low dose oral birth control must also use a barrier method of contraception for the duration of the study - WOCBP who are non-lactating and have been using an effective form of birth control for a minimum of 6 months prior to screening and agree to continue using the effective form of birth control during the study and for 30 days after the treatment period. - Male patients who are sterile or agree to use an approved method of contraception, with an approved method of contraception considered a barrier method plus a contraceptive jelly. If the female sexual partner of the male is using an approved form of contraception or is not of child-bearing potential, the male is not required to be sterile or, if not sterile, to use an approved form of contraception. - Patients who are capable of providing informed consent and complying with all study procedures/requirements. | - Presence of any uncontrolled systemic disease (e.g. cardiovascular disease, hypertension, diabetes mellitus etc.) - Presence of musculoskeletal or neurological disorders - Regular consumption of any analgesic or anti-inflammatory drug(s), either prescription or non-prescription - Taking any medications known to affect protein metabolism (e.g. b blockers, corticosteroids, non-steroidal anti-inflammatory, or prescription strength acne medications). Additional medications and/or supplementation aids (e.g. whey protein) will be deemed inappropriate based on the investigator’s discretion. - Recent (within 6 months) treatment with anabolic steroids. - Dietary conditions - Diet restrictions including vegetarianism, veganism, soy-free diet, Fish and/or fish oil allergy or intolerance - Milk allergy excluding lactose intolerance, Follows a kosher diet. - Participation in clinical trials/human studies within 1 month of the start of this study and received other medicines or has applied other medical devices. - Medications - Use of appetite stimulants (including megestrol, dronabinol and cyproheptadine), If participant is already taking a fish oil supplement, he/she must be willing to either stop the supplement. - Person who is expected to receive other medicines or applied medical devices by participating in other clinical trials/human studies during the participation period in this study. - Women who plan to become pregnant during the study, are pregnant or lactating, or were pregnant or nursing within the past year - Patients who have any condition or showing symptoms of a condition that would make them, in the opinion of the Investigator, unsuitable for the study. - Additional - Patient has implantable device such as a pacemaker or ICD - Hospitalization within the last 30 days, Living in a skilled nursing facility or long-term care facility |

**Supplementary Table 2.** Adverse events

| **System Organ Class  Preferred Term** | **Placebo (N=40)** | **Treatment (N=40)** |
| --- | --- | --- |
|  | **E(%)** | **E(%)** |
| No. Of TEAE | 7(17.50) | 10(25.00) |
| **Gastrointestinal Disorders** | 1(2.50) | 4(10.00) |
| Constipation | 0(0) | 1(2.50) |
| Nausea | 1(2.50) | 3(7.50) |
| **General disorders and administration site conditions** | 2(5.00) | 3(7.50) |
| Pyrexia | 2(5.00) | 3(7.50) |
| **Infections and infestations** | 2(5.00) | 0(0) |
| Nasopharyngitis | 2(5.00) | 0(0) |
| **Nervous system disorder** | 2(5.00) | 2(5.00) |
| Headache | 2(5.00) | 2(5.00) |
| **Respiratory, thoracic and mediastinal disorders** | 0(0) | 1(2.50) |
| Cough | 0(0) | 1(2.50) |

* Data are number of adverse events. [E] - no. of AEs experienced by n patient. TEAE; Treatment Emergent Adverse Event
